# Release Assessment Methodology for Safe, Sustainable, and Recyclable By-Design Practices for Plastics: The Epoxy–Resin Composite Case Study

**DOI:** 10.3390/nano16070403

**Published:** 2026-03-27

**Authors:** Virginia Cazzagon, Patrizia Marie Schmidt, Bastien Pellegrin, Herve Fontaine, Delphine Tissier, Arrate Huegun, Valeria Berner, Carl-Christoph Höhne, Sebastien Artous, Socorro Vázquez-Campos, Camilla Delpivo

**Affiliations:** 1 LEITAT Technological Center, C/de la Innovació, 2, 08225 Terrassa, Spain; vcazzagon@leitat.org (V.C.); svazquez@leitat.org (S.V.-C.); 2 BASF SE, Carl-Bosch-Str. 38, 67056 Ludwigshafen, Germany; patrizia-marie.schmidt@basf.com; 3 University Grenoble Alpes, CEA, Liten, Department of New Materials Technologies, 38000 Grenoble, France; bastien.pellegrin@cea.fr (B.P.); herve.fontaine@cea.fr (H.F.); sebastien.artous@cea.fr (S.A.); 4 IPC—Plastic and Composite Industrial Technical Center, 3 rue Emile Duclaux, 63360 Saint Beauzire, France; delphine.tissier@ct-ipc.com; 5 CIDETEC, Basque Research and Technology Alliance (BRTA), Po. Miramón 196, 20014 Donostia-San Sebastian, Spain; ahuegun@cidetec.es; 6 Fraunhofer Institute for Chemical Technology ICT, Joseph-von-Fraunhofer-Str. 7, 76327 Pfinztal, Germany; valeria.berner@ict.fraunhofer.de (V.B.); carl-christoph.hoehne@ict.fraunhofer.de (C.-C.H.)

**Keywords:** release assessment, Safe and Sustainable by Design, plastics, composite, microplastics, nanoplastics, environmental risk assessment, substances of concern, emerging pollutants

## Abstract

The development of new materials that are inherently safe and sustainable has become a critical objective in the context of the green transition. This challenge is especially significant for plastics, which often contain complex mixtures of chemicals that may be released during various stages of their life cycle and that can pose risks to human health and the environment. Within this context, the Safe and Sustainable by Design (SSbD) framework was followed to support the design of an innovative epoxy–vitrimer composite that integrates non-releasable fire-retardant functionalities, aiming to produce safer, sustainable, and recyclable materials suitable for railway applications. A simple methodology was developed to identify release hotspots potentially affecting workers, consumers, and environmental species and organisms. Based on this, experimental simulations were conducted to evaluate the release of materials such as flame retardants, non-intentionally added substances, and microplastics at hotspots and to compare release profiles between a benchmark material and an SSbD alternative. The results demonstrate that the newly developed recyclable and less hazardous composites can also reduce material release under weathering and abrasion conditions.

## 1. Introduction

During the last years, a lot of effort has been made to implement the Safe and Sustainable by Design (SSbD) Framework developed by the European Commission (EC) Joint Research Centre (JRC) [1], where the definition of criteria and evaluation procedures for chemicals [2], the application of SSbD framework to case studies [3], and the SSbD methodological guidance [4] still lack of specific methodologies to assess releases along the life cycle of the product development. The use of standardized and reliable communication tools should be further investigated to improve/overcome data and information gaps [5]. Moreover, the application of SSbD in real production contexts is still a key point for the refinement of the Framework, as guidance on how and when to apply existing methodologies, as well as assessment tools are needed for pragmatic and flexible SSbD implementation [6]. Close attention is paid in the context of plastic production, where plastic contamination is a key point in the current EU regulations, such as the Zero Pollution Action Plan and the EU Circular Economy Action Plan. Indeed, over the past twenty years, even more advanced investigations were conducted about the widespread presence and unintentional release in the environment of unreacted starting substances, residual processing aids, impurities, and reaction by-products with a specific focus on substances of concern, such as microplastics, generated from the degradation of plastic products [7,8,9,10,11]. As a result, in October 2023, an EU regulation under REACH was published that restricts the sale of primary microplastics, both as standalone substances and as intentionally added components to products: the EC 2023/2055 [12]. Regulation of unintentionally released secondary micro- and nanoplastics (MNPs) is not yet established, but it may become increasingly important with the introduction of the Ecodesign for Sustainable Products Regulation. Understanding the release of microplastics along a product’s lifecycle allows designers and manufacturers to reduce release at the source through material selection, product design, and process changes. Accurate data on release rates and mechanisms are also essential for risk assessment, regulation, and for prioritizing interventions that protect ecosystems and human health. Moreover, quantifying secondary microplastic emissions helps ensure that circular-economy measures (e.g., recycling, reuse) do not unintentionally increase microplastic pollution, enabling more genuinely sustainable product systems.

For this reason, in the Horizon Europe SURPASS project, a Safe, Sustainable and Recyclable by Design integrated approach has been developed and tested in three composite materials case studies, representing three key market sectors: construction, automotive, and packaging. How the Safe, Sustainable, and Recyclable by Design (SSRbD) approach has been made and tested in case studies through an interdisciplinary group of experts is presented in Soeteman-Hernandez et al. 2025 [13], where a tailor-made guidance for identifying polymer material relevant information is described.

However, while the hazard assessment as Step 1 of the JRC SSbD framework is operational and well-structured through the H classification, methodologies to easy identify hotspots of released materials and transformed substances at the early stage of the product development are missing, especially for complex materials such as plastics and their composites, where many different species can be released along the product’s life cycle. This represents a critical gap, since without robust approaches to anticipate where and how release or emissions may occur along the life cycle, it is difficult to implement (re)design measures, compare alternative materials and production routes, or assess the safety of emitted substances for workers, consumers, and the environment.

For this reason, in the current work, a methodology to assess the release of materials to support the Step 2 and 3 of the SSbD Framework implementation for composite materials is presented. Moreover, we demonstrate its implementation in a real case study: an epoxy–resin composite material containing halogen-based flame retardants to be potentially used as the exterior part of a train. Halogen-based agents are bromine- or chlorine-based compounds used to reduce flammability in plastics, electronics, insulation, and textiles. Some examples include Tetrabromobisphenol A (TBBPA), Hexabromocyclododecane (HBCD), Chlorinated Paraffins, and Tri(3-chloropropyl) phosphate (TCPP). These substances have been commonly used as flame retardants, but they are being phased out due to concerns regarding toxicity, persistence in the environment, and bioaccumulation. For this reason, the release assessment of these substances is of high importance to improve the materials design, avoiding any release along the life cycle. The assessment started with the identification of material release hotspots along the lifecycle. For this purpose, a template based on European Chemicals Agency (ECHA) guidance and scientific publications was developed to collect the data and information on substances, activities, and corresponding scenarios that were proven to influence release or emissions. A traffic-light classification was then applied to flag potential release hotspots. Next, actual releases at those hotspots were quantified experimentally by simulating the relevant activities. Finally, a scoring-system was developed and applied to determine whether the SSRbD alternative achieved improved release performance compared with the reference material, as reported in Artous et al. submitted [14]. The contribution of our work towards the development of new functional plastic composites is to provide a fast-screening methodology to assess the release of different materials during their production and use. The objective and the scientific purpose of this article is to give support to the design of a material/product by providing methodologies that allow investing less time and money at this early stage of innovation. At later stages of development, at higher technology readiness level (TRL), more advanced experimental methods will be needed to deeply characterize released substances with more sophisticated and expensive analytical techniques.”

## 2. Materials and Methods

### 2.1. Case Study Description

To increase the safety, sustainability, and recyclability performances of the metal material used in the exterior structural part of a train, different SSRbD strategies were selected and implemented. The replacement of a metal material with a plastic composite one would allow a significant reduction in vehicle weight and, thus, energy consumption during its use. Moreover, the use of non-halogenated flame retardants (FRs) contributes to the improvement of human health and environmental safety.

**Figure 1:**
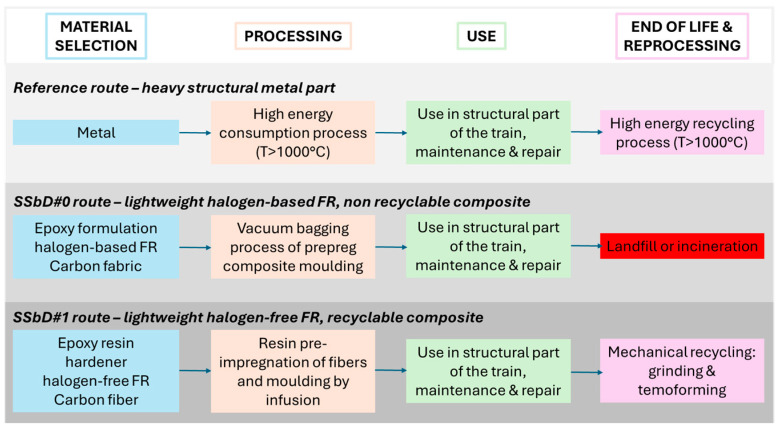
Reference and SSbD routes (SSbD#0 and SSbD#1).

Figure 1 shows the starting point used in this case study (reference route), in which the metal part is expected to be substituted by a light-weight material in order to decrease energy consumption during use in railway applications and recycling at the end of life. A promising alternative emerging in the last year is represented by the Safe and Sustainable by Design route (indicated hereafter as SSbD#0) using a lightweight material (carbon fibre epoxy composite), which is, however, not recyclable and contains a halogen-based FR. To go further, a halogen-free FR is used, combined with a reversible hardening technology, where the infusion manufacturing process permits the development of a recyclable composite, thus increasing the performance in terms of safety and sustainability (indicated hereafter as SSbD#1 route). To investigate the release performances of the different SSbD implemented strategies, SSbD#0 and SSbD#1 have been tested.

The SSbD#0 route is represented by a composite containing the halogen-based FR Tris(1-chloro-2-propyl) phosphate (TCPP) (i.e., Araldite LY1564, Huntsman Advanced Materials, Guangzhou, China and TCPP). The composite material representing the SSbD#1 route is SP3 RTM, which is made by a flame-retardant epoxy resin (i.e., Araldite LY1564 and Exolit EP360, Clariant Plastics & Coatings, Frankfurt am Main, Deutschland, respectively) and carbon fibres. Both composites were fabricated through the resin transfer manufacturing process. To investigate the contribution of carbon fibres in the material release during simulated exposure scenarios of SP3 RTM, a composite without carbon fibres was also tested (indicated as SP3). All the details related to the chemistry behind the tested materials are described in Berner et al., 2025 [15].

### 2.2. Release Assessment Methodology

Considering the consequential relationship between release and exposure, the proposed methodology aims to investigate release to inform designers who can modify their materials to minimize it, minimizing, as a consequence, the possible exposure of target populations [16,17,18]. For exposure to occur, the released materials must be emitted into an environmental compartment where they can undergo fate and transformation processes (transmission) before coming into contact with, and potentially entering the body of the receptor(s) through a specific exposure route [19,20,21]. For this reason, the proposed methodology is based on (i) the release hotspots identification; (ii) quantification of actual released materials through experimental work when a hotspot of release is identified; and (iii) a scoring system strategy to verify if the SSbD alternative has better performance in terms of release than the reference material, which is included in Artous et al. submitted [14].

### 2.3. Release Hotspots Identification

To identify potential release of substances/materials along the life cycle of each case study (from the manufacturing to the end-of-life, and eventually considering re-use), knowledge acquired in previous projects as well as scientific publications dealing with the identification of hotspots of release were considered, such as the work performed in screening safety assessment in [22,23,24] or the investigations related to transformation processes included in [25,26,27], ECHA guidelines [28,29,30,31]. An Excel file was developed to organize important information related to the substances used and the activities performed at each stage of the life cycle.

The release/emission hotspot identification was performed in a simple and pragmatic way, since this first step should be kept as easy and accessible as possible, to allow the different actors in the value chain (all those people/entities involved with different roles from material/product idea and design, to production, processing, distribution and final use) to perform this check as a preliminary step of the exposure assessment. In our approach, a release or emission hotspot was defined by the concomitant presence of a combination of variables related to both the material or product under study, the process/activity that was performed with it, and some relevant system-related parameters. As a reference, material intrinsic properties such as the presence of known toxic elements, the physical state (e.g., powder), the size (e.g., fine or ultrafine particles), or the shape (e.g., fibre or elongated particles) of some of the components of the material, can play an important role in the release of materials. Moreover, high-energy activities (e.g., spray, high-energy mixing or grinding, etc.) are associated with a likely release or emission of materials. Finally, system-related parameters (e.g., humidity, presence of containment system, ventilation, engineering controls, etc.) are known to affect material emissions. For this reason, the above-mentioned parameters were included in an Excel file (accessible through Zenodo 10.5281/zenodo.17817147), and their presence and/or combination were then used to flag possible release/emission hotspots to be further investigated using exposure assessment models or experimental testing.

Expert judgement—meaning judgement from scientists with a high level of knowledge and extensive experience (more than 10 years) in the field of emission and release assessment—and experiences acquired in previous EU projects (i.e., GUIDEnano, caLIBRAte, GRACIOUS, CIMPA, SAbyNA, ASINA, SbD4Nano) were used to identify the hotspots of release, in case of data gaps.

The experimental set-ups were designed to mimic those scenarios in which a likely hotspot of release was identified. The characterizations to be performed were selected following information found in the literature about the type of material/chemical potentially released (e.g., powder and fibre-like particles, micro-nanoplastics (MNPs)), as well as potential transformation processes occurring during the assessment scenarios [32].

In Table 1, the likely hotspots of release are presented for each Assessment Scenario.

In the current work, only the sequential ageing and abrasion experiments, simulating the use of the composite material as an exterior structural part of the train, were reported. During the use stage, powder of the polymeric composite, micro and nano fibres, and MNPs can be released [33,34] as a result of cracking and fragmentation after exposure to Xenon light [35,36,37,38,39,40].

As fibres play a dominant role in terms of performance on aged samples [41,42], a focus on released fibre-like particles was conducted. Indeed, according to the fibre (pathogenicity) paradigm [43,44] and as stated by the World Health Organization, respirable fibres with a length > 5 µm, a respirable diameter < 3 µm and an aspect ratio (A/R) > 3:1 are regarded as critical fibres as they can penetrate deep into the alveolar region of the lungs and may cause severe harm to humans upon inhalation exposure if they are sufficiently bio-durable [45]. Moreover, respirable fibres with a diameter greater than 30 nm can be classified as rigid fibres and are considered more hazardous than non-rigid fibres [46]. Information related to the hazardous effects of the hardener used in this case study can be found in Berenguer et al. (submitted).

### 2.4. Release Quantification at Identified Hotspots

#### 2.4.1. Outdoor Ageing and Hard Abrasion Test

To simulate the use stage of these materials, cascade activities (i.e., two or more tests repeated sequentially) were applied to investigate the contribution of sunlight, rain, and strong abrasion once the composite is used as an exterior part of a train.

A climatic chamber SUNTEST XXL (Atlas Material Testing Technology GmbH, Linsengericht, Germany) has been used to simulate outdoor UV ageing conditions following ISO4892-3:2014 [47,48]. Samples were taken out of the ageing device at different time slots (0–500 h, 750 h) for abrasion testing and corresponding physico-chemical characterization.

For the abrasion, a Taber Abrader with 2 wheels of 1 kg each, covered with a medium abrasive paper, has been used to simulate a strong and heavy abrasion (500 cycles) without using the aspiration system of the instrument. The thickness and weight of samples were measured after each abrasion; the fragments generated were collected for SEM observations.

Inductively Coupled Plasma-Mass Spectrometry (ICP-MS) analysis has been conducted using the triple quadrupole ICP-QQQ (Agilent 8900, Santa Clara, CA, USA) after a total digestion to analyze the Sulphur (S) element to track the 4-Aminophenyldisulfide (AFD) hardener in each investigated sample.

Scanning Electron Microscopy (SEM) JEOL J-7100FE (JEOL (Europe) BV, Lireweg, The Netherlands), operating at 20 keV with a secondary electron detector, has been used to study the powder generated during the abrasion. The specimens were coated with carbon to increase their conductivity. Manual measurement of particle size was performed using ImageJ software v1.54. More details on materials, instruments, and testing performed are reported in Supplementary Information.

#### 2.4.2. Micro- and Nanoplastic Spot-Check

In the microplastic spot-check analysis, the NanoRelease sampling protocol was applied before and after 2000 h of UV ageing of the test specimen. The purpose of the NanoRelease protocol is to remove loosely attached secondary micro- and nanoplastics from the surface of the (aged) test specimen and to disperse them in water for subsequent analysis [49]. Three different techniques were used for subsequent analysis of released micro- and nanoplastics: (1) A particle counter was used to quantify the number of released µm-sized fragments from 1 to 120 µm. (2) Analytical ultracentrifugation (AUC) was used to quantify the mass of released nanoplastics from 20 to 999 nm. (3) The release of water-soluble non-particulate organics (DOC) was performed via filtration and total organic carbon (TOC) measurements. More detailed information on the experimental set-ups, specimens used, sampling techniques, and measurements performed is reported in the Supplementary Information.

## 3. Results and Discussion

### 3.1. Release Quantification at Identified Hotspots

#### 3.1.1. Outdoor Ageing and Hard Abrasion

The effect related to the sequential release experiments of hard abrasion and outdoor ageing is presented in terms of weight loss of the sample, thickness, and elemental analysis of the powder released during abrasion.

**Figure 2:**
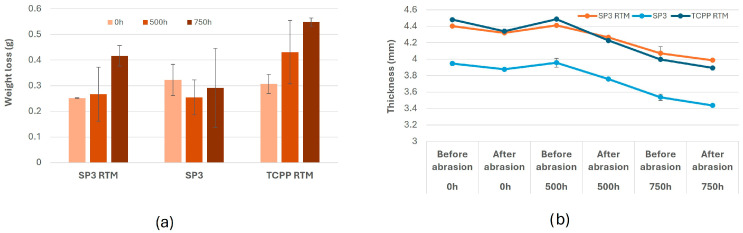
Weight loss (in g) of each sample after abrasion at 0 h, at 500 h, and 750 h of ageing (**a**) and thickness measurements (mm) of aged and not aged samples before and after the abrasion (**b**). All ageing and release experiments were performed in duplicate, and variability is represented as the difference between independent replicates.

For the weight loss (Figure 2a), it is possible to notice differences between the effect of the abrasion alone (represented as the weight loss at 0 h) and the effect of the combination of abrasion and weathering (i.e., at 500 h and 750 h). Higher variability of the weight loss after the weathering of SP3 RTM, SP3, and TCPP RTM between the two repetitions was observed compared to the variability of the samples before weathering, probably due to the effects of water and light on samples that probably compromise the sample surface, creating a non-homogeneous surface with weaker points that release more materials when subjected to the hard abrasion. In addition, no significant differences can be noticed between the SSbD1 composite (i.e., SP3 RTM) and its corresponding version without carbon fibres (i.e., SP3). Higher release of material after weathering and abrasion of the benchmark material (i.e., TCPP RTM) can be observed compared to the SSbD alternative (i.e., SP3 RTM). A reduction in the thickness of each material can be observed after the abrasion, both in weathered and non-weathered samples (Figure 2b). Lower values can be detected for samples without carbon fibres (i.e., SP3) compared to the ones containing carbon fibres (i.e., SP3 RTM, TCPP RTM), and a progressive decrease in the thickness along the ageing for all the samples. Moreover, after ageing, a slight thickness increase was detected, probably due to the effect of the water and the light during the 500 h ageing, which could alter the structure of each sample.

Considering the release investigations by elemental analysis expressed as wt. % (Table 2), sulphur—element used to track AFD release—is released in higher quantities from samples with no carbon fibres (i.e., SP3) compared to the one containing the fibre material (i.e., SP3 RTM, TCPP RTM) during the hard abrasion test. This is because the carbon fibres’ functionality improves the stability and the resistance of the composite samples. Similar sulphur concentrations are obtained following the abrasion of SP3 RTM6 and TCPP RTM1.1, which does not permit discrimination of the release of AFD between the SSRbD alternative (halogen-free FR) and the benchmark composite (halogenated FR) during the simulation of the use stage. Similar releases between weathered and non-weathered samples can be detected, showing that the weathering does not influence the AFD release during the use stage of the various materials.

**Figure 3:**
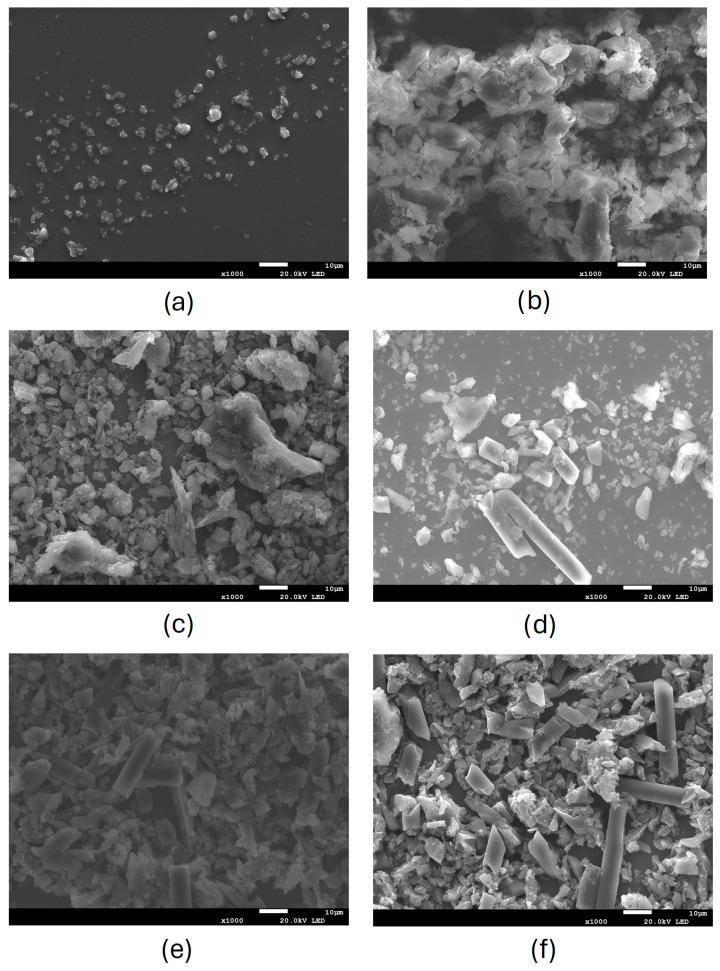
SEM images of powder released after abrasion of (**a**) SP3 at 0 h, (**b**) SP3 at 500 h, and (**c**) SP3 at 750 h, (**d**) SP3 RTM at 0 h, (**e**) SP3RTM at 500 h, and (**f**) SP3 RTM at 750 h.

**Figure 4:**
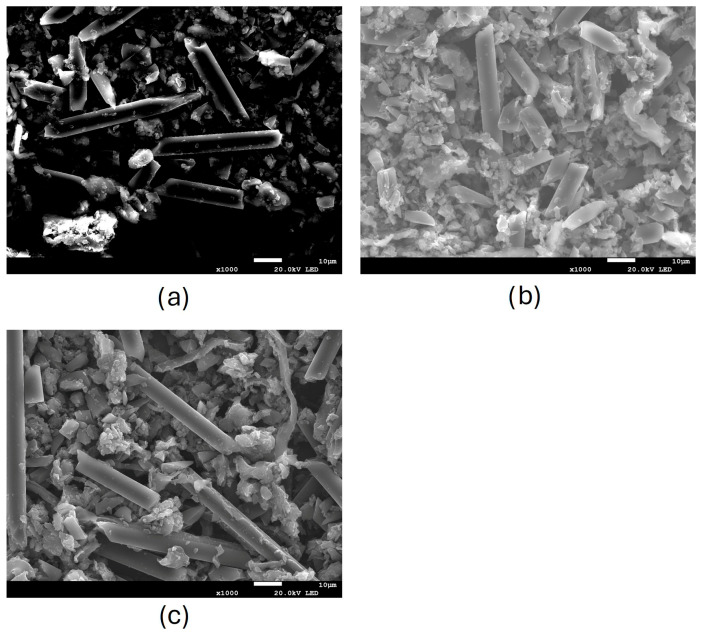
SEM images of powder released after abrasion of TCPP at (**a**) 0 h, (**b**) 500 h, and (**c**) 750 h.

According to SEM observations (Figure 3 and Figure 4), the particles collected after the abrasion of non-aged and aged materials have a quite broad size distribution, characterized by particles having irregular spheroidal shapes. In samples containing the carbon fibre materials (SP3RTM and TCPP RTM), fibre fragments have different lengths.

After weathering, particles seem to be more aggregated, and the determination of the particle size is more challenging due to the presence of overlapping particles with very similar contrast in the SEM images. This increased aggregation makes it more difficult to measure small-sized particles and results in an apparent larger particle size.

Regarding the fibres released from the composites containing the carbon fibre materials (RTM), in all observed samples, the fibre diameter was 6.5 ± 0.5 µm, which can be considered as rigid fibres. The fibre diameter did not change after the first abrasion and the subsequent weathering plus abrasion, indicating that these types of fibres are not affected by these processes. Regarding the length of fibres, very few of them were shorter than 5 µm after the first abrasion, and their length remained quite constant after the subsequent weathering plus abrasion. Accordingly, the fibres A/R remained higher than 3:1. The number of fibres per picture and the number of fibres with A/R > 3, for aged and not aged samples, were quite similar, suggesting that the SSbD alternative (i.e., SP3 RTM) has the same behaviour when subjected to the combined weathering and abrasion. In Table 3, the measurements for length, diameter, and the percentage of fibres with an A/R higher than 3 are reported for 15 fibres of each sample, where the highest value was obtained for the TCPP material abraded and aged for 750 h.

#### 3.1.2. Outdoor Ageing and Micro- and Nanoplastic Quantification

**Figure 5:**
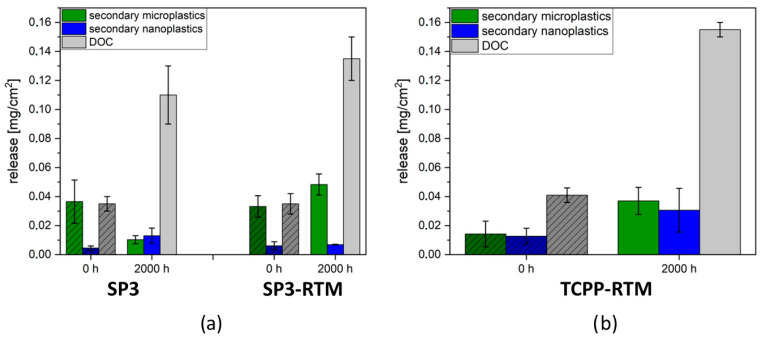
Release of secondary microplastics, nanoplastics, and DOC in mg/cm^2^ from the SSbD alternatives (**a**) and the reference (**b**) before and after UV ageing. All ageing and release experiments were performed in duplicate, and variability is represented as the difference between independently incubated replicates.

Each species of SP3 with and without carbon fibres (SP3 RTM and SP3, respectively) and the TCPP Reference with carbon fibres (TCPP RTM) was measured in duplicate. The measured raw data obtained with the three different analytical techniques were used to calculate the release of each species in mass per (aged) surface area. Figure 5a shows the comparison of releases from the SP3 (without carbon fibres) and the SP3-RTM (with carbon fibres) material, each pristine and aged. Figure 5b shows the releases from the TCPP-RTM Reference pristine and aged. After UV ageing, the DOC release is increased 3-4-fold in SP3, as well as in SP3-RTM. In the case of SP3, the microplastic release is decreased after UV ageing, and the nanoplastic release only slightly increased. In contrast to that, an increase in micro- and nanoparticle release from the SP3-RTM composite material was detected after UV ageing. In this context, it must be noted that micro- and nanoparticles released from the composite materials are most likely composite fragments as well. However, due to the definition of microplastics, they all must be classified as microplastics if their polymer content is at least 1%, which is very likely the case, but can currently not be proven with the available analytical techniques. In the case of the TCPP-RTM Reference without carbon fibres, an increase in micro-, nanoplastic, and DOC release was detected after UV ageing (Figure 5b). Particle releases from pristine TCPP-RTM Reference samples were lower compared to pristine SP3 samples (with and without carbon fibres). The nanoparticle release from the TCPP-RTM Reference detected after 2000 h UV ageing is, however, higher than the nanoparticle release from SP3 and SP3-RTM after the same ageing duration. Compared to the pristine TCPP-RTM, the micro- and especially the nanoparticle release increased after ageing.

Comparing the Reference material (i.e., TCPP-RTM) and the SSRbD alternative material (i.e., SP3-RTM), the microparticle release from the Reference was, in most cases, lower than from the SSRbD product, but the nanoparticle release was, especially after ageing, higher than the nanoparticle release from any other SSRbD material (pristine and aged).

Overall, the comparative analysis between the SSbD alternative (SP3 RTM) and the benchmark material (TCPP RTM) revealed distinct differences in release behaviour under simulated use and ageing conditions. The SSbD composite consistently demonstrated reduced material release during both weathering and abrasion scenarios compared to the benchmark containing halogenated flame retardant, suggesting enhanced durability and resistance to environmental stressors. Notably, the release of MNPs from the SSbD composite was generally lower or comparable to the benchmark, particularly in the nanoparticle fraction after ageing, which is critical, given the heightened concern over nanoparticle emissions.

The obtained results, therefore, underscore the effectiveness of the SSbD approach in achieving its core objectives: minimizing hazardous releases, enhancing material recyclability, and supporting safer product innovation. By integrating non-halogenated flame retardants and reversible hardening technologies, the SSbD composite not only meets functional performance requirements for railway applications but also addresses key sustainability and safety criteria. The systematic identification and quantification of release hotspots throughout the product life cycle demonstrate how SSbD methodologies can proactively guide material selection and process optimization. The observed reduction in material release, especially under stress conditions that mimic real-world use, aligns with the SSbD framework’s emphasis on source reduction in emissions and supports regulatory trends such as the EU’s restrictions on microplastics and the upcoming Ecodesign for Sustainable Products Regulation. Importantly, the findings highlight the value of early-stage release assessment in the innovation process, enabling the anticipation and mitigation of potential risks before large-scale deployment.

For workers, the identification of release hotspots during manufacturing and end-of-life stages (e.g., fibre handling, grinding) provides insights for exposure mitigation, including technical measures and personal safety equipment. The SSbD composite’s lower propensity for releasing hazardous fibres and particles during high-energy processes directly translates to reduced inhalation and dermal exposure risks. From an environmental perspective, the decreased release of MNPs, as well as other substances of concern, supports ecosystem protection and aligns with circular economy principles by ensuring that recycling and reuse do not inadvertently increase pollution.

## 4. Conclusions

Our work contributed to the development of new plastic composites made of halogen-free flame retardant, providing a fast-screening methodology to assess the release of materials during their production and use stages. The contribution of our work towards the development of new functional plastic composites is to provide a fast-screening methodology to assess the release of different materials during their production and use. The objective and the scientific purpose of this article is to give support to the design of a material/product by providing methodologies that allow investing less time and money at this early stage of innovation. At later stages of development, at higher TRL, more advanced experimental methods will be needed to deeply characterize released substances with more advanced methods and corresponding analytical techniques (e.g., Pyrolysis–Gas Chromatography–Mass Spectroscopy or Raman for MNPs [50]). This approach is meant to be in line with the SSbD framework implementation, where all the safety and sustainability investigations should reflect the TRL of the material/product/process assessed.

This study presents a preparatory work for the development of a more structured methodology for assessing material release within the SSbD framework, specifically applied to an epoxy–resin composite intended for railway applications. By integrating expert knowledge, regulatory guidance, and experimental validation, the approach enables early identification of release hotspots across the product life cycle—from manufacturing to end-of-life. The developed Excel-based template for release hotspot identification, grounded in ECHA guidelines, facilitated a systematic screening of potential release scenarios. Experimental simulations of identified hotspots at the use stage allowed quantification of particles, fibres, and MNPs emissions. Comparative analysis between the benchmark and the SSRbD alternative demonstrated that the newly developed recyclable composites can also reduce material release, especially under weathering and abrasion conditions.

The findings underscore the importance of incorporating release assessments early in material innovation to guide safer and more sustainable design choices, especially to investigate releases of Substances of Very High Concern (SVHC) and their transformation processes along the life cycle of a product. Moreover, the results provide a foundation for developing a scoring system that integrates release data with hazard profiles, enabling a more holistic SSbD evaluation.

Future work will focus on refining the scoring methodology, validating the approach across broader material types, and exploring its integration into regulatory and industrial decision-making processes. Ultimately, the developed methodology provides a transparent, reproducible methodology to inform regulatory alignment, safer substitution, and data-driven material selection, contributing to the advancement of SSbD practices by offering a pragmatic tool for anticipating and mitigating environmental and human exposure risks associated with plastic-based materials.

## Figures and Tables

**Table 1:** Hotspots of release were identified, and an associated experimental test was performed.

Life Cycle Stage	Activity	Potential Hotspot of Release	Potential Receptors of Release/Exposure	Substance of Interest	ER/EC	Experimental Test
Step 1 and Step 2—Material design (Formulation and manufacturing)—FR resin formulation	Handling/cutting of fibres	YES—dry solid carbon fibres, high energy level of the process, manual activity, 60’of duration	Workers	Micro and nanofibres	Inhalation, dermal	NA
Step 3—Use	Use as an exterior structural part of the train	YES—potential release during ageing and abrasion	Environment	Powder of the composite, micro and nano fibres, MNPs	Soil, water	Outdoor ageing and hard abrasion
Step 4—Mechanical recycling	Grinding	YES—dry micro composite particles/powder and/or carbon fibres, high energy level of the process, manual activity	Workers	Powder of the composite, micro and nano fibres, MNPs	Inhalation, dermal	NA

ER/EC: Potential exposure route(s)/exposed environmental compartments, NA: activity excluded from the study, MNP: micronanoplastics.

**Table 2:** S content (% *w*/*w*) after total digestion of a 1 × 1 cm^2^ sample and corresponding total S concentration calculated for the abraded volume sample.

Sample	S (% *w*/*w*)—1 × 1 cm^2^ Sample (Mean ± SD)	S (% *w*/*w*) Related to the Total Abraded Sample Volume (Mean ± SD)
SP3RTM	1.73 ± 0.06	51.92 ± 1.77
SP3	6.30 ± 0.11	188.92 ± 3.22
TCPP RTM	1.53 ± 0.01	46.05 ± 0.26

**Table 3:** Number of fibres detected in SEM images and percentage of elongated particles with aspect/ratio (A/R) higher than 3 for the samples containing carbon fibres.

Sample	Weathering	Diameter (µm)	% Elongated Particles with A/R > 3
TCPP RTM	0 h	5.92 ± 0.88	53%
	500 h	6.75 ± 0.52	60%
	750 h	6.99 ± 0.72	73%
SP3 RTM	0 h	6.64 ± 0.49	60%
	500 h	6.77 ± 0.32	53%
	750 h	6.77 ± 0.55	67%

## Data Availability

The Excel file developed in the study is openly available in Zenodo at https://doi.org/10.5281/zenodo.17817147.

## Supplementary Materials

The following supporting information can be downloaded at: https://www.mdpi.com/article/10.3390/nano16070403/s1, Figure S1. Hard abrasion and weathering, respectively, using the (a) Taber abrader and the (b) SUNTEST XXL+ climatic chamber, and (c) the effect of 750 h of weathering on the samples. Figure S2. Equipment used to measure the thickness of the composite samples. Refs. [47,48] are cited in the supplementary materials.

## Abbreviations

The following abbreviations are used in this manuscript:

A/R	Aspect ratio
AFD	4-Aminophenyldisulfide
AUC	Analytical ultracentrifugation
DOC	Dissolved Organic Carbon
EC	European Commission
ECHA	European Chemicals Agency
FR	Flame retardants
HBDC	Hexabromocyclododecane
IAS	Intentionally added substances
ICP-MS	Inductively Coupled Plasma- Mass Spectrometry
JRC	Joint Research Centre
MNPs	Micro- and nanoplastics
NIAS	Non-intentionally added substances
SEM	Scanning Electron Microscopy
SSbD	Safe and Sustainable by Design
SSRbD	Safe, Sustainable, and Recyclable by Design
SVHC	Substances of Very High Concern
TBBPA	Tetrabromobisphenol A
TCPP	Tris(1-chloro-2-propyl) phosphate
TOC	Total Organic Carbon
TRL	Technology Readiness Level
