# Supplementary material for: Release Assessment Methodology for Safe, Sustainable, and Recyclable By-Design Practices for Plastics: The Epoxy–Resin Composite Case Study
Source: Nanomaterials (Basel). 2026 Mar 27;16(7):403. doi: 10.3390/nano16070403 (PMC13075047; doi:10.3390/nano16070403)
Supplement: Supplementary file 1 [file nanomaterials-16-00403-s001.zip › nanomaterials-4201827-supplementary.pdf]

Release assessment methodology for safe, sustainable, and recyclable by-design practices for plastics: the epoxy–resin composite case study

Virginia Cazzagon [1], Patrizia Marie Schmidt [2], Bastien Pellegrin [3], Herve Fontaine [3], Delphine Tissier [4], Arrate Huegun [5], Valeria Berner [6], Carl-Christoph Höhne[6], Sebastien Artous [3], Socorro Vázquez-Campos [1], Camilla Delpivo [1*]

Supporting Information. Hard abrasion following outdoor ageing

<u>Ageing and abrasion experiment</u>

A climatic chamber SUNTEST XXL (ATLAS Electrical Devices) has been used to simulate outdoor UV ageing conditions. Specifically, daylight filters and Xenon lamps have been used following Method A of ISO4892:2024 [47]. Up to 2000 h of accelerated aging, or nearly 3 months in the laboratory simulator, accumulated 432MJ/m2UV energy, which corresponds to roughly two years under mid-European external conditions [48]. In this study, samples were taken out of the aging device at different time slots (0-500 h – 750 h) for abrasion testing and corresponding physico-chemical characterisation. The weathering was stopped at 750h of aging as the samples already showed a strong morphological alteration, e.g., possible oxidation due to the UV effect. Duplicates of each composite were prepared with a size of 10 x 10 cm for the cascade activity, while samples of 1 x 1 cm of size were prepared to analyse the total Sulphur content.

For the abrasion, a Taber Abrader with two wheels of 1 kg each, covered with a medium abrasive paper, has been used to simulate a strong and heavy abrasion (500 cycles) without using the aspiration system of the instrument. The thickness of each sample at three different points of the abraded surface was measured before and after abrasion (after removing the powder obtained from the abrasion) using the micrometre Mitutoyo model S1012XB. Thickness measurements were presented as mean $\pm$ standard deviation of three independent measurements. An analytical balance (Denver Instrument, APX-200) has been used to weigh samples and wheels with the sanding paper before and after performing abrasion. Statistical significance was assessed using confidence intervals calculated for duplicate measurements to compare sample weights after identical weathering durations. Results are reported in grams with four decimals for the mean and standard deviation of the two replicates of each material.

The thickness of each sample at three different points was measured before and after abrasion (after removing the powder obtained from the abrasion) using the micrometre Mitutoyo model S1012XB (Figure S2). Results are reported in millimetres with two decimals as mean and standard deviation between the two replicates of each material. An analytical balance (Denver Instrument, APX-200) has been used to weigh samples and wheels with the sanding paper before and after performing abrasion. Results are reported in grams with four decimals for the mean and standard deviation of the two replicates of each material.

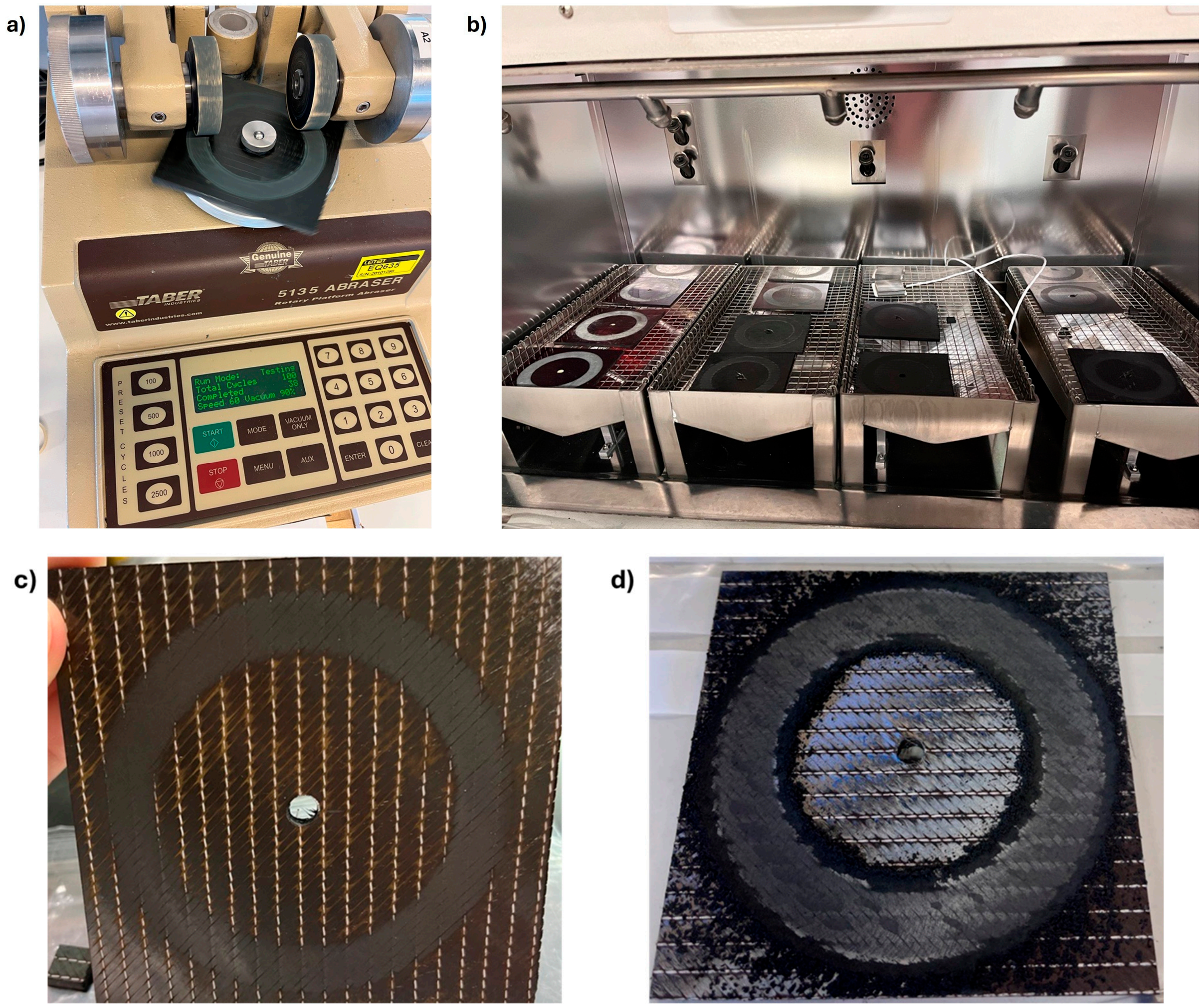

Figure S1. Hard abrasion and weathering, respectively, using the a) Taber abrader and the b) SUNTEST XXL+ climatic chamber, and c) the effect of 750h of weathering on the samples.

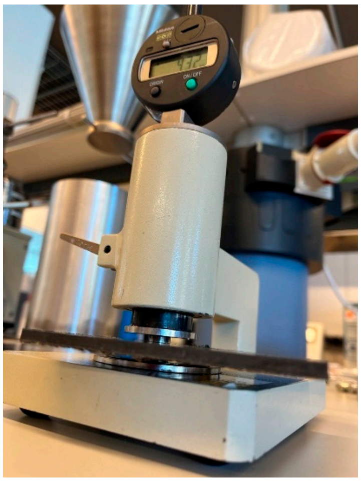

Figure S2. Equipment used to measure the thickness of the composite samples.

<u>Inductively Coupled Plasma-Mass Spectrometry</u>

Inductively Coupled Plasma-Mass Spectrometry (ICP-MS) analysis has been conducted using the triple quadrupole ICP-QQQ (Agilent 8900) to analyse the Sulphur (S) element to

track the AFD hardener in each investigated sample (i.e., clean sanding paper as a blank, sanding paper after abrasion (left and right wheel paper separately) and composite samples 1 x 1 cm size before weathering). A triple quadrupole ICP-QQQ (Agilent 8900) was used after a total digestion of each investigated sample with 4 mL of concentrated ultrapure nitric acid (HNO3 70 %) and 1 mL of concentrated ultrapure hydrochloric acid (HCl 37 %) in an analytical microwave at 280 ° C, and each weighted sample was analysed before the digestion. Samples are weighed in an analytical balance (Denver Instrument, APX-200) with accuracy +/- 0.0001 g directly in the digestion vials. The digestion residue obtained is then diluted appropriately to analyse Sulphur (S). Quantification of this element is performed by interpolation on a calibration line prepared from commercial standards of the elements of interest. The test is performed in duplicate, and results are expressed as mean ± standard deviation of the independent measurements.

The following samples were processed using the above-mentioned protocol:

- Clean sanding paper as a blank.
- Sanding paper after abrasion (left and right wheel paper separately).
- Composite samples 1 x 1 cm in size before weathering.

Scanning Electron Microscopy

Scanning Electron Microscopy (SEM) JEOL J-7100FE, operating at 20 keV with a secondary electron detector, has been used to study the powder generated during the abrasion. The powder generated from the abrasion was collected with a few drops of milliQ water and deposited on carbon tape attached to SEM stubs to determine particle morphology and size distribution of the material released. The specimens were coated with carbon to increase their conductivity. Manual measurement of particle size was performed using ImageJ software. Spheroidal and fibre particles were counted and measured separately. At least 100 particle measurements were performed, and results are reported as mean ± standard deviation of the diameter in µm and the percentage of fibres-like particles present in each sample.

Micro- and nanoplastic spot-check

The pristine and aged specimen subjected to the NanoRelease sampling protocol had all the same surface dimensions of 6 cm2. For sampling, 6 mL of ultrapure water was placed in a glass container, and one specimen was placed onto it (with the aged side facing the water). In the next step, the sample was treated in the ultrasonic bath at 100% power for 1 hour. During this time, regular cooling with ice was necessary to maintain the temperature below 40°C. After the ultrasonication treatment, the plate was removed, and the liquid was collected using a syringe. The collected volumes, the initial volume used for the treatment, and the sizes of the specimens were documented. Blank measurements in the absence of a specimen were conducted to determine the particle background. Three different techniques were used for subsequent analysis of released micro- and nanoplastics, as well as dissolved organic carbon (DOC): a particle counter, analytical ultracentrifugation (AUC), and part of the solution was filtered < 0.02 µm to assess the release of water-soluble non-particulate organics (DOC) via TOC measurements.

The particle counter measurements were performed with an Abakus® Mobil Fluid laser particle counter (Klotz GmbH) equipped with a laser sensor LDS 2148(0) (1-120 µm). 0.7 mL of the NanoRelease dispersion was filled up to 500 mL with ultrapure water. For sensor protection, the sample was filtered with a 190 µm pre-filter (nylon, Rotert). The measurement was performed with 200 mL of the sample. Data evaluation was performed with C.A.R. Lab. Duplicate samples were measured, and values for blank measurements were subtracted. Based on the measured counts and size distributions of released microplastics with a known density, the numbers were converted into a mass, assuming a spherical particle shape.

The AUC-Beckman XL centrifuge is equipped with an interference optical system synchronised to the centrifugal rotation, so that the colloidal sedimentation profile during centrifugation is monitored over time. By adjusting the centrifugation speed, the mass concentration and particle size distribution were determined in three overlapping size intervals of 10-150 nm (12 000 rpm ≙ 10 500 rcf), 40-800 nm (3 000 rpm ≙ 650 rcf), and 300-5000 nm (1 000 rpm ≙ 70 rcf), with 3 hours centrifugation time each. For data evaluation of the absorption profiles (resolved in time and radius), SedFit v14.0 was used with the corresponding polymer density. Duplicate samples were measured, and values for blank measurements were subtracted.

The release of water-soluble non-particulate organics (DOC) was performed via TOC measurements: the filtered (cut off < 0.02 µm) sample was diluted with ultrapure water (dilution factors of 1:10 and 1:20) and total carbon (sample combusted catalytically on a Pt/Al2O3 catalyst in an oxygen stream at ca. 680 °C) and total inorganic carbon (sample acidified with 25 wt.% phosphoric acid) were quantified as CO2 via NDIR detection (Shimadzu TOC-L). DOC was calculated as the difference between total carbon and total inorganic carbon. Duplicate samples were measured.
